# HIV testing among United States high school students at the state and national level, Youth Risk Behavior Survey 2005–2011

**DOI:** 10.1186/2193-1801-3-202

**Published:** 2014-04-24

**Authors:** Karen Coeytaux, Michael R Kramer, Patrick S Sullivan

**Affiliations:** Episight Consulting, 10 Garden Road, Summit, NJ USA; Department of Epidemiology, Rollins School of Public Health, Emory University, Atlanta, GA USA

**Keywords:** HIV testing, Adolescent, High school, Risk behavior, YRBS

## Abstract

**Background:**

Human immunodeficiency virus (HIV) remains an important public health issue and CDC recommends routine HIV screening for Americans aged 13–64. Adolescents and young adults are disproportionately affected compared to the overall population. We analyzed self-reported HIV testing and related risk behaviors at the state and national level among youths who had sexual intercourse, with a focus on state level differences.

**Methods:**

This study used the state and national Youth Risk Behaviors Surveys 2005–2011. It included a total of 59,793 national-level observations and 39,421 state-level observations of US high school students, of which respectively 28,177 and 13,916 reported ever having sexual intercourse.

The outcome of interest was having ever been tested for HIV. The risk behaviors were condom use at last intercourse, number of sexual partners in lifetime, age at first intercourse, ever forced sexual intercourse, and ever illegal injection drug use. Analyses performed included logistic regression and *t*-test analyses.

**Results:**

HIV testing was positively associated with HIV-related risk behaviors among sexually active high school students. However, HIV testing remained relatively low (22%) between 2005 and 2011, even for those engaging in risk behaviors. Results differed among the only 7 states that monitored HIV testing through YRBS, most commonly with respect to HIV testing and condom use.

**Conclusions:**

Routine HIV testing is critical for early identification of HIV, which was set as a priority in a recent Executive Order. Our data suggest further efforts are needed to achieve widespread uptake of HIV testing among high school students. Furthermore, differences observed across states likely reflect different needs and should be followed up closely by states. Finally, having accurate data that reflects the reality of youths’ lives is crucial for efficient prevention planning. Thus, more states should consider collecting HIV testing data to evaluate uptake of HIV testing among youth.

## Background

Human Immunodeficiency Virus (HIV) remains an important public health issue among young people in the United States. Compared to the overall population, adolescents and young adults are disproportionately affected by HIV. Youth aged 13–24 represented 4% of all diagnosed cases by the end of 2010, but 21% of new diagnoses in 2011 (CDC 2011).

As recommendations have moved toward earlier treatment, early testing is getting more attention as a gateway to care. Benefits of early diagnosis of HIV infection are multiple (CDC 2013b). It provides patients with timely access to treatment and thus improves their quality of life and survival time. People, who initiate antiretroviral therapy before getting to a CD4 count below 350 cells per μL, have a significantly reduced risk for AIDS-related events or death (Sterne et al. 2009). Early HIV diagnosis is also crucial in limiting the spread of the disease in the general population. Infected patients becoming aware of their positive HIV status can adapt their behavior to avoid infecting others, and engage in HIV treatment, which substantially reduces their infectiousness (Branson et al. 2013).

It is now clear that control and elimination of HIV will be possible only with widespread testing, prompt and accurate diagnosis, and universal access to antiviral therapy (Branson et al. 2013). Also, as patients are likely to be asymptomatic at early stage of HIV infection, prompt diagnosis implies routine screening.

In 2006, the Center for Disease Control and Prevention (CDC) recommended routine voluntary HIV screening in all individuals aged 13 to 64 regardless of recognized risk factors (Branson et al. 2006). In line with these recommendations, the United States Preventive Services Task Force now recommends that clinicians screen all individuals aged 15–65 for HIV (Moyer 2013). This implies that an important proportion of adolescents should be routinely tested for HIV infection, regardless of their risk level. Recently, the President issued an Executive Order emphasizing the national priority of improving all phases of the HIV care continuum, including early HIV diagnosis (Gardner et al. 2011; The White House 2013).

Still, at the end of 2009, one in five (18%) US adults and adolescents living with HIV infection was unaware of its status. Youth aged 13–24 are estimated to be the age group with the highest share of people unaware about their HIV infection. In 2009, 76,400 aged 13–24 were estimated to be living with HIV and six out of ten were undiagnosed (CDC 2010). Many adolescents still have a limited knowledge about HIV/AIDS and underestimate their personal infection risk (Gurvey et al. 2005). One of the main limitations to self-initiated testing among adolescents is the concern about confidentiality (Hyden et al. 2014). The inadequate access to HIV prevention and treatment services was also identified as a major barrier, as some adolescents have only limited contact with the health care system (Irwin et al. 2009). Finally, the implementation of routine HIV testing in the real-life can remain challenging and in some locations it is still sometimes only offered to patients perceived at high risk (Jain et al. 2009).

Moreover, diagnosis often occurs late in the course of the disease. During 2010, people aged 13–24 represented 12.7% of persons with stage 3 (AIDS) classification at the time of HIV diagnosis. Furthermore, additional efforts are needed to systematically link newly diagnosed individuals to treatment and reduce drop offs across the continuum of HIV care (Gardner et al. 2011). Thus, legislation changes under the Affordable Care Act aiming at extending HIV care coverage represent an important step forward (The White House 2013).

Understanding testing patterns of youth is essential in better scaling up HIV testing. The National HIV Behavioral Surveillance System enrolls people aged 18 or more who are in identified high risk groups, and thereby does not reflect testing patterns of younger people (Gallagher et al. 2007). Thus, the Youth Risk Behavioral Survey (YRBS), which collects data from a representative sample of high school students, can be used as an alternative to understand current patterns of HIV testing among youths.

A previous report demonstrated the association of HIV testing with some risk behaviors using the 2009 national YRBS (Balaji et al. 2012). To update this previous report and further address gaps in knowledge, we analyzed YRBS over the period 2005–2011, at the state and national levels. We explored further the association between HIV testing and HIV-related risk behaviors, including state level differences, because such differences may reflect different testing needs across states. The present report includes, (i) estimates of HIV testing and selected risk behaviors among youths who had sexual intercourse, (ii) associations between HIV testing and selected behaviors associated with HIV acquisition (HIV risk behaviors), (iii) comparison of state and national estimates of HIV testing and risk behaviors.

## Results

Of 59,793 high school student observations from the national YRBS for the period 2005–2011, 28,177 reported prior sexual intercourse. The distributions of sexually active students across sex and grade levels were similar for each survey year. Only one out of five students reported ever testing for HIV. Most students did not engage in studied HIV-related risk behaviors (Table 1).

**Table 1:** **Characteristics of High School Students Who Had Sexual Intercourse, National YRBS 2005–2011**

Characteristic	Students, % (95% CI) (n = 28177)
Sex of the subject	
Male	52.1 (51.3–52.9)
Female	47.9 (47.1–48.7)
Grade of the subject	
9th grade	19.8 (18.9–20.6)
10th grade	23.7 (23.0–24.4)
11th grade	26.8 (26.1–27.5)
12th grade	29.7 (28.9–30.6)
Race and ethnicity	
White	55.6 (52.4–58.8)
Black or African American	18.7 (16.6–21.1)
Hispanic	18.7 (16.8–20.7)
Other	6.9 (5.9–8.1)
Ever been tested for HIV	
Yes	21.9 (21.0–22.8)
No, not sure	78.1 (77.2–79.0)
Use of condom at last sexual intercourse	
No	35.4 (34.3–36.4)
Yes	64.6 (63.6–65.7)
4 or more sexual partners in life	
Yes	31.1 (30.1–32.0)
No	68.9 (68.0–69.9)
First sexual intercourse before 13	
Yes	13.5 (12.8–14.2)
No	86.5 (85.8–87.2)
Ever forced to have sexual intercourse	
Yes	14.1 (13.4–14.8)
No	85.9 (85.2–86.6)
Lifetime illegal injection drug use	
Yes	3.5 (3.2–3.9)
No	96.5 (96.1–96.8)

*Abbreviations:* *HIV* human immunodeficiency virus.

Crude analysis showed that the odds of HIV testing were higher for females compared to males, for students in 12th grade compared to students in lower grades and for Black or African American students compared to other race and ethnicity groups. Moreover, the odds of HIV testing were higher for students engaging in HIV-related risk behaviors (Table 2).

**Table 2:** **Characteristics Associated With HIV Testing Among Students Who Had Sexual Intercourse, National YRBS 2005–2011**

	Students, No. (n = 28177)	cOR (95% CI)	aOR (95% CI)
Sex of the subject			
Male	14463	Referent	See Interaction
Female	13614	1.72 (1.59–1.86)	See Interaction
Grade of the subject			
9th grade	4749	Referent	See Interaction
10th grade	6147	1.10 (0.96–1.26)	See Interaction
11th grade	7944	1.19 (1.04–1.36)	See Interaction
12th grade	9154	1.39 (1.22–1.58)	See Interaction
Race and ethnicity			
White	10684	Referent	See Interaction
Black or African American	6834	1.67 (1.45–1.92)	See Interaction
Hispanic	8106	1.00 (0.90–1.12)	See Interaction
Other	2085	1.17 (1.01–1.36)	See Interaction
Use of condom at last sexual intercourse			
Yes	17614	Referent	See Interaction
No	9919	1.63 (1.51–1.76)	See Interaction
Four or more sexual partners in life			
No	18771	Referent	See Interaction
Yes	9116	2.59 (2.40–2.79)	See Interaction
First sexual intercourse before 13			
No	24077	Referent	See Interaction
Yes	3966	1.67 (1.51–1.85)	See Interaction
Ever forced to have sexual intercourse			
No	24156	Referent	See Interaction
Yes	3856	2.24 (2.03–2.47)	See Interaction
Lifetime illegal injection drug use			
No	26630	Referent	Referent
Yes	930	2.51 (2.07–3.04)	1.68 (1.26–2.25)
2-way interactions			
**Race and Number of sexual partner in life**			
White			
Less than 4 sexual partners in life	7750	Referent	Referent
4 or more sexual partners in life	2883	2.86 (2.55–3.20)	2.50 (2.21–2.82)
Black or African American			
Less than 4 sexual partners in life	3769	Referent	Referent
4 or more sexual partners in life	2968	1.80 (1.58–2.05)	1.73 (1.48–2.02)
Hispanic			
Less than 4 sexual partners in life	5612	Referent	Referent
4 or more sexual partners in life	2395	2.20 (1.86–2.61)	1.99 (1.66–2.39)
Other			
Less than 4 sexual partners in life	1354	Referent	Referent
4 or more sexual partners in life	699	2.95 (2.27–3.83)	2.72 (2.06–3.58)
**Sex and Age at first sexual intercourse**			
Male			
First sexual intercourse at 13 or later	11465	Referent	Referent
First sexual intercourse before 13	2918	2.26 (1.97–2.60)	1.42 (1.21–1.66)
Female			
First sexual intercourse at 13 or later	12531	Referent	Referent
First sexual intercourse before 13	1033	1.49 (1.27–1.75)	0.99 (0.83–1.18)
**Sex and Use of condom at last sexual intercourse**			
Male			
Use of condom at last sexual intercourse	9890	Referent	Referent
No use of condom at last sexual intercourse	4178	1.24 (1.10–1.40)	1.07 (0.93–1.22)
Female			
Use of condom at last sexual intercourse	7673	Referent	Referent
No use of condom at last sexual intercourse	5701	1.76 (1.57–1.97)	1.48 (1.32–1.65)
**Grade and Ever forced sexual intercourse**			
9th			
No forced sexual intercourse	3970	Referent	Referent
Ever forced sexual intercourse	748	2.14 (1.66–2.77)	1.35 (1.05–1.72)
10th			
No forced sexual intercourse	5227	Referent	Referent
Ever forced sexual intercourse	881	2.21 (1.78–2.73)	1.60 (1.28–2.00)
11th			
No forced sexual intercourse	6847	Referent	Referent
Ever forced sexual intercourse	1059	2.77 (2.29–3.35)	1.93 (1.57–2.37)
12th			
No forced sexual intercourse	7974	Referent	Referent
Ever forced sexual intercourse	1126	1.93 (1.61–2.31)	1.24 (1.02–1.50)

*Abbreviations:* *cOR* unadjusted OR, *aOR* adjusted odds ratio, *HIV* human immunodeficiency virus.

In the logistic regression model, four interaction terms were significant. First, although HIV testing was more prevalent among students with 4 or more sexual partners for all race and ethnicity groups, it was associated with higher odds of testing among White and Other race students than among Black/African American or Hispanic students. Second, males with first sexual intercourse before 13 had higher odds of HIV testing compared to males with first sexual intercourse at 13 or later. No significant difference was found for females. Third, females who did not use a condom at last intercourse were more likely to be tested for HIV compared to females who used a condom at last sex. No significant difference was found for males. Fourth, the odds of HIV testing were the highest among 11th graders, when comparing students ever forced to have intercourse to those never forced (Table 2).

Of 39,421 high school student observations from the states’ YRBS for the period 2005–2011, 13,916 reported prior sexual intercourse. For each of the seven states with available data, the distribution of high school students who had sexual intercourse was comparable across sex and grade levels. Still, Connecticut, New Jersey, and North Dakota had slightly greater proportions of students in 12th grade compared to 9th grade. Racial and ethnic composition varied substantially across states. Most students had never been tested for HIV and never engaged in HIV-related risk behaviors but with cross-state differences (Table 3).

**Table 3:** **Characteristics of High School Students Who Had Sexual Intercourse by State, State YRBS 2005–2011**

Characteristic	MA 2005–2011 (n = 4884) % (95% CI)	CT 2007–2011 (n = 2342) % (95% CI)	NJ 2005,07,11 (n = 1972) % (95% CI)	AR 2009–2011 (n = 1282) % (95% CI)	NC 2009–2011 (n = 3434) % (95% CI)	ND 2009–2011 (n = 1445) % (95% CI)	SC 2009 (n = 529) % (95% CI)
Sex of the subject							
Male	51.7 (49.9–53.5)	51.3 (48.8–53.9)	51.7 (48.7–54.8)	49.9 (45.7–54.0)	51.2 (48.9–53.5)	48.4 (45.1–51.7)	51.0 (46.1–56.0)
Female	48.3 (46.5–50.1)	48.7 (46.1–51.2)	48.3 (45.2–51.3)	50.1 (46.0–50.3)	48.8 (46.5–51.1)	51.6 (48.3–54.9)	49.0 (44.0–53.9)
Grade of the subject							
9th grade	17.8 (15.4–20.4)	15.2 (11.6–19.6)	14.7 (11.7–18.3)	19.8 (13.0–29.1)	20.7 (16.5–25.6)	14.4 (10.8–19.0)	24.3 (16.1–35.0)
10th grade	22.3 (19.6–25.2)	19.7 (16.8–23.0)	20.7 (16.7–25.3)	25.3 (20.4–30.9)	23.8 (20.1–27.9)	22.5 (17.7–28.2)	23.7 (15.0–35.2)
11th grade	28.0 (24.8–31.5)	28.2 (23.5–33.5)	28.1 (23.8–32.8)	26.7 (21.5–32.6)	27.9 (22.4–34.1)	28.2 (22.4–34.8)	23.9 (17.2–32.2)
12th grade	31.9 (28.1–36.0)	36.9 (33.1–40.8)	36.5 (30.9–42.6)	28.2 (23.0–34.0)	27.7 (22.7–33.2)	34.8 (28.8–41.4)	28.1 (20.8–36.7)
Race and ethnicity							
White	69.1 (64.1–73.6)	62.3 (55.6–68.5)	54.8 (45.8–63.4)	65.0 (56.0–73.1)	51.0 (43.8–58.1)	80.0 (73.6–85.2)	49.0 (38.4–59.7)
Black or African American	10.4 (8.0–13.4)	16.3 (13.1–20.0)	19.6 (13.5–27.7)	25.8 (18.0–35.5)	36.1 (29.3–43.5)	0.8 (0.5–1.2)	44.4 (33.8–55.5)
Hispanic	15.1 (12.6–18.0)	18.2 (14.4–22.7)	21.6 (17.2–26.8)	6.6 (4.9–8.9)	6.6 (5.5–8.0)	2.1 (1.6–2.9)	3.1 (2.4–4.1)
Other	5.5 (4.5–6.6)	3.2 (2.7–3.9)	4 (3.26–4.9)	2.5 (2.0–3.3)	6.2 (4.4–8.8)	17.0 (12.0–23.6)	3.4 (2.4–4.8)
Ever been tested for HIV							
Yes	21.6 (19.4–24.0)^a^	22.1 (19.3–25.1)	18.9 (16.1–22.1)^a^	26.4 (23.5–29.5)	23.0 (20.8–25.3)	23.6 (21.4–26.0)	19.6 (15.7–24.1)
No, not sure	78.4 (76.0–80.6)^a^	77.9 (74.9–80.7)	85.8 (77.9–83.9)^a^	73.6 (70.5–76.5)	77.0 (74.7–79.2)	76.4 (74.0–78.6)	80.4 (75.8–84.3)
Use of condom at last sexual intercourse							
No	36.0 (34.1–38.0)	35.0 (32.4–37.6)	30.1 (27.3–33.1)	39.8 (35.9–43.8)	38.8 (36.6–41.0)	38.5 (35.7–41.4)	36.5 (30.5–42.9)
Yes	64.0 (62.0–65.9)	65.0 (62.4–67.6)	69.9 (66.9–72.7)	60.2 (56.2–64.1)	61.2 (59.0–63.4)	61.5 (58.6–64.3)	63.5 (57.1–69.5)
4 or more sexual partners in life							
Yes	27.7 (25.56–30.0)	26.6 (24.6–28.8)	28.4 (25.1–31.9)	36.0 (32.7–39.5)	32.6 (29.9–35.5)	28.0 (25.3–30.9)	36.6 (32.2–41.2)
No	72.3 (70.0–74.4)	73.4 (71.2–75.4)	71.6 (68.1–74.9)	64.0 (60.5–67.3)	67.4 (64.5–70.1)	72.0 (69.1–74.7)	63.4 (58.8–67.8)
First sexual intercourse before 13							
Yes	11.4 (10.3–12.5)	12.0 (10.0–14.3)	9.7 (8.2–11.5)	17.7 (14.0–22.1)	15.4 (13.6–17.3)	7.8 (6.4–9.6)	17.3 (13.9–21.2)
No	88.6 (87.5–89.7)	88.0 (85.7–90.0)	90.3 (88.5–91.8)	82.3 (77.9–86.0)	84.6 (82.7–86.4)	92.2 (90.4–93.6)	82.7 (78.8–86.1)
Ever forced to have sexual intercourse							
Yes	16.6 (15.5–17.8)	13.4 (12.4–14.5)	13.7 (11.8–15.8)	16.3 (13.4–19.7)	14.1 (12.3–16.1)	11.8 (9.9–13.9)	14.3 (11.1–18.2)
No	83.4 (82.2–84.5)	86.6 (85.5–87.6)	86.3 (84.2–88.2)	83.7 (80.3–86.6)	85.9 (83.9–87.7)	88.2 (86.1–90.1)	85.7 (81.8–88.9)
Lifetime illegal injection drug use							
Yes	3.0 (2.4–3.7)	4.2 (2.8–6.3)	2.4 (1.9–3.0)	3.4 (2.29–4.9)	2.1 (1.4–3.4)	3.2 (2.2–4.5)	2.1 (1.2–3.7)
No	97.0 (96.3–97.6)	95.8 (93.7–97.2)	97.6 (97.0–98.1)	96.6 (95.–97.7)	97.9 (96.6–98.6)	96.8 (95.5–97.8)	97.9 (96.3–98.8)

^a^HIV testing estimates for the period 2007–2011 in Massachusetts and 2009–2011 in New Jersey. *Abbreviations:* *HIV* human immunodeficiency virus, *MA* Massachusetts, *CT* Connecticut, *NJ* New Jersey, *AR* Arkansas, *NC* North Carolina, *ND* North Dakota, *SC* South Carolina.

**Figure 1:**
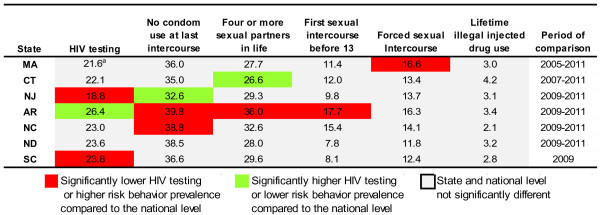
**State and National Prevalence Comparison of HIV Testing and HIV-Risk Behaviors, State and National YRBS.** The comparison of state and national prevalence of HIV testing and engagement in risk behaviors among high school students who had sexual intercourse, while controlling for sex, grade, and race and ethnicity are presented in this figure. Abbreviations: HIV, human immunodeficiency virus; MA, Massachusetts; CT, Connecticut; NJ, New Jersey; AR, Arkansas; NC, North Carolina; ND, North Dakota; SC, South Carolina. ^a^HIV testing estimates for the period 2007–2011.

The comparison of state and national prevalence of HIV testing and engagement in risk behaviors among high school students who had sexual intercourse, while controlling for sex, grade, race and ethnicity, revealed some significant differences (Figure 1). The prevalence of HIV testing among sexually active students in Arkansas was higher than the national average, as opposed to New Jersey and South Carolina, where it was below the national average. Compared to the national level, Arkansas and North Carolina had a higher prevalence of students who did not use condom at last intercourse. Also, Arkansas had a higher prevalence of students who had four or more partners in life and of students who had their first sexual intercourse before 13. Finally, Massachusetts had a higher prevalence of students with forced intercourse. Conversely, compared to the national average, New Jersey had significantly lower prevalence of students who did not use condom at last intercourse and Connecticut had a significantly lower prevalence of students who had four or more partners in life.

## Discussion

This study showed that HIV testing among students who had sexual intercourse remained relatively low (22%) and stable (data not shown) between 2005 and 2011, despite CDC’s 2006 recommendations for making HIV testing a standard part of the medical care for individuals aged 13–64 (Branson et al. 2006). These results were consistent with the literature (CDC 2012). Furthermore, this study highlighted that HIV testing and risk behavior profiles differ across states. HIV testing differences could be interpreted in the context of risk behaviors. This new piece of information is important for future prevention interventions among youths.

Our results confirmed that HIV testing was more common among females, Black or African Americans, 12th graders, and students engaging in HIV-related risk behaviors (Balaji et al. 2012). The three risk factors most strongly associated with HIV testing were having four or more lifetime sexual partners, lifetime illegal injection drug use, and ever having been forced to have sexual intercourse. This was consistent with previous studies (Arrington-Sanders et al. 2008; Balaji et al. 2012; Decker et al. 2005, 2014; Miller 2010; Samet et al. 1997; Talib et al. 2013). However, Swenson reported higher odds of rapid HIV testing among those with only one sex partner in the past 90 days than those with multiple partners, using a sample of 81 adolescents (Swenson et al. 2011). Observed differences are likely due to smaller sample size (Swenson et al. 2011). The association of HIV testing with lifetime illegal injection drug use and ever having been forced to have intercourse were also reported among young adults (Decker et al. 2009; Kellerman et al. 2002; Williams-Roberts et al. 2010).

Several interaction terms were found to be significant in this study, adding nuance to previous findings (Balaji et al. 2012). Large size in YRBS provides more statistical power for the detection of interactions.

### Interaction between race and number of sexual partners

Although number of sexual partners was significantly associated with testing for all students, it was particularly important for White students. One possible explanation could be the higher level of HIV testing among Black or African American students with less than 4 partners compared to White and Hispanic. This could also be explained by the lower use of condom among high school students with higher numbers of sexual partners, in particular among White, as reported by Richter et al. (1993).

### Interaction between sex and age at first intercourse

Having first intercourse before 13 was associated with higher odds of HIV testing among males. One possible explanation could be that the share of black students is higher among males who had sex before 13 compared to males who had sex after 13, whereas it is similar for females who had sex either before or after 13 (Cavazos-Rehg et al. 2009). As Black students have higher rates of HIV testing, this could drive the difference between the two male subgroups.

### Interaction between sex and use of condom

Not using a condom at last intercourse was associated with higher odds of HIV testing among females compared to males. Young women, who use condoms as contraception and did not use one at last sex, are likely to get tested for pregnancy at a care provider (Daley et al. 2005) and should receive HIV testing, given efforts for integrating pregnancy and STIs prevention among teens (Tran et al. 2010). Besides, young women who did not use condom at last sex, but use oral contraception, are likely to receive counseling and HIV testing from the doctor who prescribes their contraceptive (American College of Obstetricians and Gynecologists (ACOG) 2008).

The comparison between states and the national sample highlighted geographic differences in HIV testing and risk behavior profiles, with the most common difference in prevalence use of condom at last sex. This reinforces previous observations of risk profile variations across locations among older adolescents (Moore et al. 2013).

These geographic differences are very informative for State Health Departments as they point toward areas that need further investments in programs. They can also inform state education agencies and school districts about local adaptations needed in HIV/AIDS educational curriculum (National Association of State Boards of Education 2013). For example, currently, only 33 states and the district of Columbia mandate HIV education and only 13 states require the inclusion of information on the possible negative outcomes of teen sex and pregnancy (Guttmacher Institute 2014). Thus, states could gain great benefits from reinforcing their programs according to the sexual risk behaviors patterns of their local youth populations.

Forty-eight states plus District of Columbia now have HIV testing laws that are consistent with the key features of the CDC’s 2006 recommendations: opt-out HIV testing, no written consent required, and no pretest counseling required (CDC 2013c). Still, some differences remain across states (e.g., minimum age required to consent, availability of anonymous testing, reporting policies of positive HIV tests) and can sometimes limit widespread HIV testing among adolescents (National HIV/AIDS Clinicians’ Consultation Center 2014). Additionally, rapidly aligning practices with new HIV testing laws can be a real challenge in itself (Hyden et al. 2014; Kelley et al. 2011). For example, some HIV locations lack a clear and consistent communication of their confidentiality policies for HIV testing due to a lack of training (Hyden et al. 2014).

This study also highlights the missed opportunity to collect HIV testing information through YRBS. Although weighted data were available for 43 states, only 7 monitored HIV testing for at least one of the years within 2005–2011. National and state use the same standard questionnaire, but state and local officials responsible for the coordination of YRBS can add or delete questions to meet their policy or programmatic needs (CDC 2013a). This can result in gaps in knowledge. Controlling HIV/AIDS epidemic requires measuring progress in HIV testing and prioritizing interventions accordingly. The comprehensive monitoring of HIV testing across all states through YRBS would be an important step forward in terms of HIV prevention among youths. Furthermore, systematic monitoring of sexual preferences would be of great value in targeting populations at increased risk of transmission, such as male having sex with male (CDC 2013d).

This study has some limitations. The cross-sectional design of YRBS prevents identification of temporal order between HIV testing and risk behaviors. Additionally, the self-reported nature of YRBS can introduce bias (e.g., recall, non-response, social desirability) (Sackett 1979). However, several studies evaluated YRBS validity and concluded to its reliability (Brener et al. 2002). Additionally, YRBS is limited to students attending high school; this results in selection bias. Finally, higher dropouts among Hispanics and Black or African American ethnicities can also raise bias issue (National Center for Education Statistics 2012).

## Conclusion

Routine HIV testing is an efficient strategy for early identification of HIV. It allows early linkage to care of HIV positive adolescents and reduces the rate of new HIV infections. Although early HIV identification became a national priority, HIV testing among high school students remains low, even for those at increased risk of HIV infection.

Moreover, state action is critical to enhance routine HIV testing among youth. Differences in patterns across states observed, are meant to be exploratory and hypothesis generating. They should be followed up by states and try to be correlated with knowledge of programs. Finally, all states should participate in YRBS to monitor accurately HIV testing and should ensure that testing laws in place are prone to the rapid and full implementation of routine HIV testing among youths.

## Methods

### Participants and procedure

This study used national and state YRBS 2005–2011. YRBS measures the prevalence of health risk behaviors among high school students, through an anonymous paper-and-pencil survey administered every other year in randomly selected public and private high schools. National and state levels are representative of high school students in the United States and states considered. They come from separate scientific samples of schools and students, follow the same survey methodology and use the same core questionnaire, to which state and local agencies can add or delete questions (CDC 2013a). National YRBS data are publicly accessible and state data are available upon request. After IRB (Emory University) approval, data were retrieved for 43 states. Data were not available for the seven remaining states due to either absence of weighted data (California, Oregon), no YRBS participation (Minnesota, Washington), or no data sharing policy (Hawaii, Indiana, and Vermont). Out of the 43 states, seven collected the information related to HIV testing through their YRBS questionnaire for at least one year in the studied period (Massachusetts: 2005–2011; Connecticut: 2007–2011; New Jersey: 2005, 2009, and 2011; Arkansas, North Carolina, North Dakota: 2009–2011; South Carolina: 2009). This study used 59,793 observations at the national level and 39,421 observations at the state level.

### Measures

#### Population of interest

The subpopulation of interest was high school students who had sex and was defined by positive answer to the question “Have you ever had sex?”.

#### Outcome variable

HIV testing was assessed through the question “Have you ever been tested for HIV, the virus that causes AIDS?”.

#### Independent variables

The five HIV-related risk behaviors selected were assessed through the questions: (i) “The last time you had sexual intercourse, did you or your partner use a condom?”; (ii) “During your life, with how many people have you had sexual intercourse?”; (iii) “How old were you when you had sexual intercourse for the first time?”; (iv) “Have you ever been physically forced to have sexual intercourse when you did not want to?”; (v) “During your life, how many times have you used a needle to inject any illegal drug into your body?”.

#### Controlling variables

Sex and grade were classified through the questions: “What is your sex?” and “In what grade are you?”. Race/ethnicity was computed from the questions “Are you Hispanic or Latino?” and “What is your race?”, and had four categories: White, Black/African Americans, Hispanic, and Other (American Indian or Native Alaskan, Asian, Native Hawaiian or other Pacific Islander, and multiracial).

### Statistical analysis

All analyses were conducted using SAS-callable SUDAAN version 9.3, accounted for the complex sampling design, and used a significant level α = 0.05. Domain analyses were used to perform calculation on the subpopulation of high school students who had sexual intercourse. Less than 10% of data were missing across all variables, except for: HIV testing in the 2011 national survey (24%) and in North Carolina (12% for 2009–2011); and lifetime illegal injection drug use in North Carolina (29% for 2009–2011). Missing data were accounted for by using the “not missing completely at random” option.

Descriptive analyses were conducted at national and state levels for the period 2005–2011. Testing prevalence, risk behavior prevalence and demographic characteristics were calculated among students who ever had sex. Finite population correction was applied for the calculation of state prevalence due to the smaller size of state samples (<5,000).

The association of HIV testing with risk behaviors at the national level for the 2005–2011 period was modeled using logistic regression. First, logistic regression was used to assess the crude association between HIV testing and each risk behavior and demographic. Multivariable logistic regression model was then used to assess the adjusted association between HIV testing and each independent variable, controlling for sex, grade, and race/ethnicity. Effect modification by sex, race/ethnicity, grade level, and year of survey was assessed through backward elimination. The Bonferroni method was used to correct multiple testing (Bland and Altman 1995). Four interaction terms were significant at the level 0.05, with k = α/4, and remained in the final model: Race/ethnicity and Number of sexual partner in life (p-value = 0.004), Sex and Age at first sexual intercourse (p-value = 0.004), Sex and Use of condom at last intercourse (p-value = 0.007), and Grade and Ever forced intercourse (p-value = 0.012). No multicolinearity was found between covariates.

State HIV testing and risk behavior prevalence were compared to national estimates. Significant differences were assessed using *t*-test controlling for sex, grade, and race/ethnicity. Multiple-testing was accounted for through a statewide significance α = 0.05 (k = α/6).

## Abbreviations

AIDS: Acquired immunodeficiency syndrome
AR: Arkansas
CDC: Center for disease control and prevention
CI: Confidence interval
CT: Connecticut
HIV: Human immunodeficiency virus
IRB: Institutional review board
MA: Massachusetts
NC: North Carolina
ND: North Dakota
NJ: New Jersey
OR: Odds ratio
SC: South Carolina
YRBS: Youth risk behavior survey.
